# Graphene Distributed Amplifiers: Generating Desirable Gain for Graphene Field-Effect Transistors

**DOI:** 10.1038/srep17649

**Published:** 2015-12-04

**Authors:** Hongming Lyu, Qi Lu, Yilin Huang, Teng Ma, Jinyu Zhang, Xiaoming Wu, Zhiping Yu, Wencai Ren, Hui-Ming Cheng, Huaqiang Wu, He Qian

**Affiliations:** 1 Institute of Microelectronics, Tsinghua University, Beijing, 100084, China; 2 Tsinghua National Laboratory for Information Science and Technology (TNList), Beijing, 100084, China; 3 Department of Electrical & Computer Engineering, University of California, San Diego, La Jolla, CA 92093, USA9; 4 Shenyang National Laboratory for Materials Science, Institute of Metal Research, Chinese Academy of Sciences, Shenyang, 110016, China

## Abstract

Ever since its discovery, graphene bears great expectations in high frequency electronics due to its irreplaceably high carrier mobility. However, it has long been blamed for the weakness in generating gains, which seriously limits its pace of development. Distributed amplification, on the other hand, has successfully been used in conventional semiconductors to increase the amplifiers’ gain-bandwidth product. In this paper, distributed amplification is first applied to graphene. Transmission lines phase-synchronize paralleled graphene field-effect transistors (GFETs), combining the gain of each stage in an additive manner. Simulations were based on fabricated GFETs whose *f*_*T*_ ranged from 8.5 GHz to 10.5 GHz and *f*_*max*_ from 12 GHz to 14 GHz. A simulated four-stage graphene distributed amplifier achieved up to 4 dB gain and 3.5 GHz bandwidth, which could be realized with future IC processes. A PCB level graphene distributed amplifier was fabricated as a proof of circuit concept.

The irreplacably high carrier mobility of graphene has been of great interests to the elctronics community and motivated researcher to endeavor on graphene radio frequency (RF) electronic devices^1^,^2^,^3^. Graphene field-effect transistors are widely studied and many research groups have generated cutoff frequencies (*f*_*T*_) of hundreds of GHz^4^,^5^,^6^,^7^,^8^,^9^, with the record-high value of 427 GHz^9^.

Poineering works on graphene circuits have led to the demonstration of frequency doublers^10^,^11^,^12^, ambipolor mixers^13^ and modulators^14^,^15^, in which graphene’s unique property of electron and hole symmetry is utilized. By superimposing an sinusoidal signal on the minimum conductance point (Dirac point), the electron or the hole branches, the frequency and phase of the output signal could be modulated. Another appliction, the graphene passive circuits (e.g. graphene resistive mixers)^16^,^17^,^18^,^19^, attracts more attentions and already shows potential to compete with conventional semiconductor technologies. The authors’ group has combined CMOS back-end-of-line (BEOL) processes and graphene to realize a two-layer-routing four-GFET double-balanced mixer IC, which generated IIP3 up to 21 dBm^19^.

However, the overall progress in electronics is greatly hindered by graphene’s gapless band structure^1^,^2^. Obviously, the lack of drain current saturation is detrimental to amplifiers. In particular, even though the *f*_*T*_ in various groups has reached hundreds GHz, the *f*_*max*_ of GFETs, representing the ability to generate power and voltage amplification, has halted at only 70 GHz^5^. Graphene researchers have tried conventional amplifier architectures. Andersson *et al.* utilized a matching surface mount inductor on the input gate to yield a return loss of 20 dB. Their work achieved S_21_ of 10 at 1 GHz^20^. Guerriero *et al.* fabricated graphene voltage amplifiers in complementary push-pull configuration, which generated a voltage gain of 11.4 dB and a −3 dB bandwidth of only 70 kHz^21^. In a recent work, a three stage cascade amplifier IC achieved a gain of 4 dB at 4.8 GHz^22^. Despite these works, graphene’s application in amplifiers with conventional topologies are mostly disappointing.

On the other hand, graphene’s IC process has witnessed substantial improvements^12^,^19^,^22^. Both Han *et al.* and the authors’ group have proposed the multi-layer-routing inverted process for graphene integration, which serves as the foundation for future novel graphene circuit architectures^12^,^19^,^22^. The inverted process utilizes CMOS BEOL processes to fabricate circuit and device structures, followed by CVD graphene transfer at the back end of the flow. This process utilizes existing CMOS technologies to the maximum extend and greatly reduces contaminations and potential damages to graphene.

In this paper, the graphene distributed amplifer is proposed to solve its long-standing weakness in amplification. Engineers have successfully applied distributed amplication to conventional semiconductor technologies to increase the amplifiers’ gain-bandwidth-product^23^,^24^,^25^,^26^, which could not be increased simply by parallelizing transistors, because the increase in the transconductance is compensated for the corresponding increase in the input and output capacitances. The distributed amplifiers place transistors along artificial transmission lines, adding the *g*_*m*_ of each transistor in a phase-synchronized manner. Therefore, the bandwidth-gain-product could be increased. While the gain of a conventional cascade amplifier is the product of the gain of each stage, distributed amplifiers’ gain is directly proprotional to the number of stages. This trait makes distributed amplification scheme especially suitable for graphene, as each GFET stage generates very modest or less-than-unity gains. The otherwise product of the gain of each stage would be mediocre.

Four GFETs with gate lengths of 300 nm through 500 nm have been fabricated, which generated *f*_*T*_ ranging from 8.2 GHz to 10.6 GHz and *f*_*max*_ 12.4 GHz to 16.6 GHz without de-embedding (Misallignment existed in this batch of devices, breaking the relationship between the *f*_*T*_ and *f*_*max*_ metrics and the gate length). The *f*_*max*_ exceeded *f*_*T*_ in these works due the low resistance of the buried gates^5^. Measured S-parameters of the GFETs were used in graphene distributed amplifier simulations. The circuit schematic employed artificial transmission lines formed by lumped inductors and capacitors to phase-syncronized each GFET stage. Simulations generated up to 4 dB gain and and 3.5 GHz bandwidth, which is the first graphene wide-band amplification dicussed in literature. These designs could be realized by IC technologies with precise models. However, at the present stage, a PCB-level distributed amplifer was fabricated. As individual GFETs and bonding wires were not precisely modeled, the passive components of any small values were avoided. Redesign of the passive components sacrificed the gain, while the bandwidth was maintained. The measured performance was in agreement with circuit simulations.

## Results and Discussion

### Graphene Field-Effect Transistors

**Figure 1:**
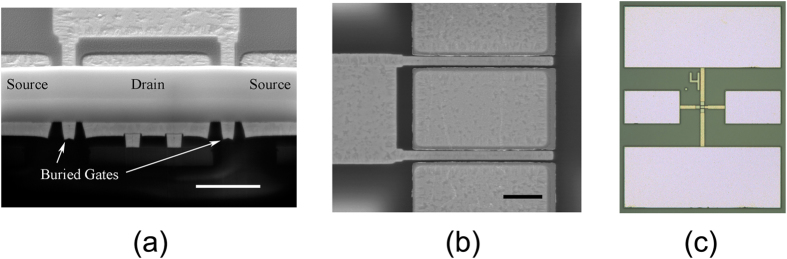
SEM and optical microscope images of the GFET. Cross section (**a**) and top (**b**) views of the GFET by SEM. Scale bars: 2 μm. (**c**) Optical microscope image of the GFET with probing pads.

GFETs in buried-gate structure have been fabricated. Cross-section and top views of a 400 nm-gate-length GFET structure are shown in Fig. 1a,b, respectively. Chemical-mechanical-planarization (CMP) process flattened the wafer surface, which guarrenteed the successfulness of the following graphene transfer process. Cross section made by focused-ion-beam (FIB) indicated the thickness of the gate of 600 nm. Such thickness effectively lowers the gate series resistance, which is favourable from *f*_*max*_ point of view. The GFETs employed a two-finger structure with each finger 6 μm wide. The gate dielectric employed HfO_2_ with equivalent oxide thickness (EOT) of 2 nm formed directly on the buried gate. Graphene was synthensized by CVD method and transferred by “bubbling” method as previously reported (details in Method)^27^,^28^. The channel of the GFETs was defined by contact photolithography and contacts by electron beam photolithography (EBL). The contacts were 40 nm Pt (details in Method).

**Figure 2:**
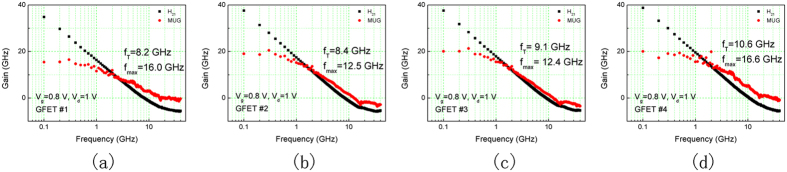
RF Performance of the GFETs. *f*_*T*_ and *f*_*max*_ of GFET #1 (**a**), GFET #2 (**b**), GFET #3 (**c**) and GFET #4 (**d**).

The probing pads were 80 μm × 80 μm with 100 μm pitch in ground-signal-ground (GSG) layout, as shown in Fig. 1c. All devices have not gone through passivation step for convenience. Several reports have shown various dielectrics, such as Si_3_N_4_^29^, BN^30^, Al_2_O_3_^31^, etc., could effectively protect graphene devices. Future works should consider passivation to increase the stability and reliability of graphene devices. S-parameters have been measured on chip up to 40 GHz under ambient atmosphere using Agilent N8230C network analyzer. Four GFETs labeled as GFET #1 ~ 4 featured gate lengths of 300 nm, 500 nm, 500 nm and 400 nm, respectively, and demonstrated *f*_*T*_*/f*_*max*_ of 8.2 GHz/16 GHz, 8.4 GHz/12.5 GHz, 9.1 GHz/12.4 GHz, 10.6 GHz/16.6 GHz, respectively, as shown in Fig. 2. These results were without de-embedding. The devices were then cut out of the chip with the probing pads which enabled wire bonding in following works.

### Distributed Amplifiers

**Figure 3:**
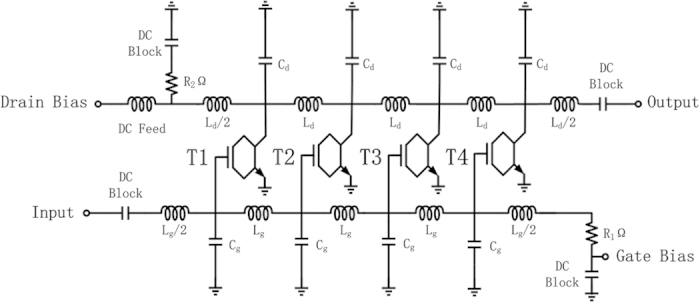
Schematic of a four-stage graphene distributed amplifier.

Figure 3 shows the schematic of a four-stage graphene distributed amplifier. Artificial transmission lines formed by lumped elements are periodically loaded with the gate and drain terminals of the GFETs, forming the so-called gate and drain transmission lines. An RF signal applied at the input end of the gate line travels down to the other end, where it is absorbed by the terminating impedance. The signals sampled by the gate of a GFET at a different location with different phases is transferred to the drain line. The two gate and drain transmission lines pocess the same phase velocity. Therefore, the gains of each stage are combined in the forward-travelling direction. The backward-traveling signal on the drain line is absorbed at the frontier end. As long as the gain per section is greater than the corresponding loss, the overall gain of a distributed amplifier can be increased even without limit. While the gain of conventional cascade amplifiers are the product of each stage, distributed amplifiers increases the gain proportional to the number of stages, which is especially attractive to graphene, since the otherwise multiplication could not generate satisfying gains. The four GFETs in the schematic are labeled as T1~4. Passive components, C_d_, C_g_, L_d_ and L_g_, constitute the gate and drain artificial lines. R_1_ and R_2_ form the two terminating resistances.

**Figure 4:**
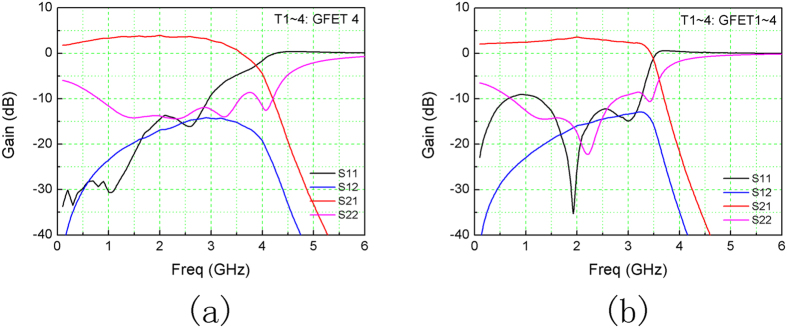
Simulation results. Magnitude of the S-parameters of Simulation #1 (**a**) and #2 (**b**).

Circuit simulation based on measured S-parameters of the GFETs was performed in a standard circuit simulator, Agilent Advanced Design System (ADS). In Simulation #1, T1 through T4 all employed GFET #4, respresenting ideal situations when GFETs are exactly identical. The optimization was performed with three goals: 1) S21 > 5 dB; 2) S11 < −10 dB; 3) S22 < −10 dB. Variables were the terminating resistances, R_1_, R_2_, and the lumped passive components, C_g_, C_d_, L_g_, L_d_ (details in Method). Source and load impedances were set as R_1_ and R_2_, respectively. Simulation #1 achieved S21 of roughly 4 dB and 3.5 GHz bandwidth, as shown in Fig. 4a. Varibles of the passive componnents are shown in Table 1. R_1_, R_2_, C_g_, C_d_, L_g_ and L_d_ resulted in 1.28 kΩ, 78.5 Ω, 0.01 pF, 0.7 pF, 105 nH and 7.6 nH, respectively. Simultion #1 represents situations when GFETs are exactly identical (T1~4: GFET #4). After the demonstration of high-performance individual GFETs in many lab works, efforts in graphene electronics society should now emphasize reproducibility and reliability to support the potential mass production.

Another simulation, Simulation #2, was launched closer to real circumstances. GFET #1 through #4 were assigned to T1 through T4, respectively. The setup was the same with Simulation #1, which generated S21 of 3 dB and 3.5 GHz bandwidth, as shown in Fig. 4b. The variables, R_1_, R_2_, C_g_, C_d_, L_g_ and L_d_ resulted in 1.28 kΩ, 58 Ω, 0.01 pF, 1.2 pF, 86 nH and 7.2 nH, respectively, as shown in Table 2, being very close to Simulation #1.

Simulation #1 and #2 for the first time demonstrated wide-band graphene amplifiers in literature. Both simulations were based on measured S-paramenters of the GFETs, illustrating the feasibilty of wide-band graphene amplifiers with present averge-performance GFETs. The passive component were subjected to the following equation that guarantees the phase-synchronization requirement:


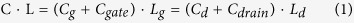


where C and L represents the capacitance and inductance at each node. For Simulation #1, C_g_, C_d_, L_g_ and L_d_ were 0.01 pF, 0.7 pF, 105 nH and 7.6 nH, respectively. Miller effects obscured C_drain_ and the gate oxide capacitance was roughly 0.08 pF, caculated according to the dimension and gate dielectric of the 400 nm GFET. C_gate_ was further decreased due to quantum capacitance effect^32^,^33^, therefore qualifying equation (1). These simulations assumed that wideband matching sections were used: the source and load impedences were set as R_1_ and R_2_, which roughly equaled characteristic impedences of the gate and drain transmission lines, respectively, caculated as 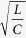. The bandwidth of a distributed amplifier is determined by the cutoff frequency of the artificial transmission line, 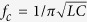
26. Following works should realize these designs by IC technology, which has a precise control of the parasitics and models of the active and passive components.

On the other hand, efforts have been made in this work to realize a PCB-level graphene distributed amplifier, which meets problems such as precise GFET models, the choice of discrete lumped passive components, and bonding wires. Any variations are likely to destroy the phase-synchronization between the gate and drain transmission lines, especially when any of the passive components values are too small, e.g., C_g_ in Simulation #1 and #2.

**Figure 5:**
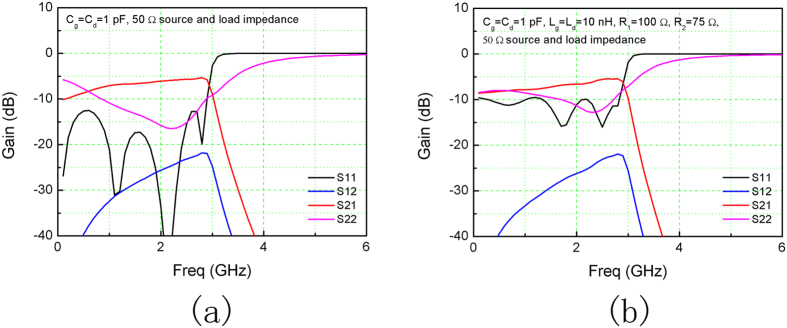
Simulation results. Magnitude of the S-parameters of Simulation #3 (**a**) and #4 (**b**).

Firstly, small values for any of C_g_, C_d_, L_g_, L_d_ are avoided in the simulations, Simulation #3 and #4. In particular, C_g_ was fixed at 1 pF (i.e., minimum value for dicrete capacitances in 0805 package). The terminating impedances were both set at 50 Ω. Simulation #3 used C_g_ = C_d_ = 1 pF and R_1_ = R_2_ = 50 Ω. The simulation result is shown in Fig. 5a, and values of the variables in Table 2. Due the large C_g_, the gain decreased to −10~−5 dB and the bandwidth remained at 3 GHz. Simulation #4 further avoided matching sections at the input and output terminals by employing 50 Ω source and load impedence. C_g_ and C_d_ were 1 pF, and L_g_ and L_d_ 10 nH, which are common discrete devices. The simulation result is demonstrated in Fig. 5b. Extraordinarily large capacitances were used in these simulations, because otherwise any variations could easily break the phase-synchronization requirement. In turn, it sacrificed the gain.

**Figure 6:**
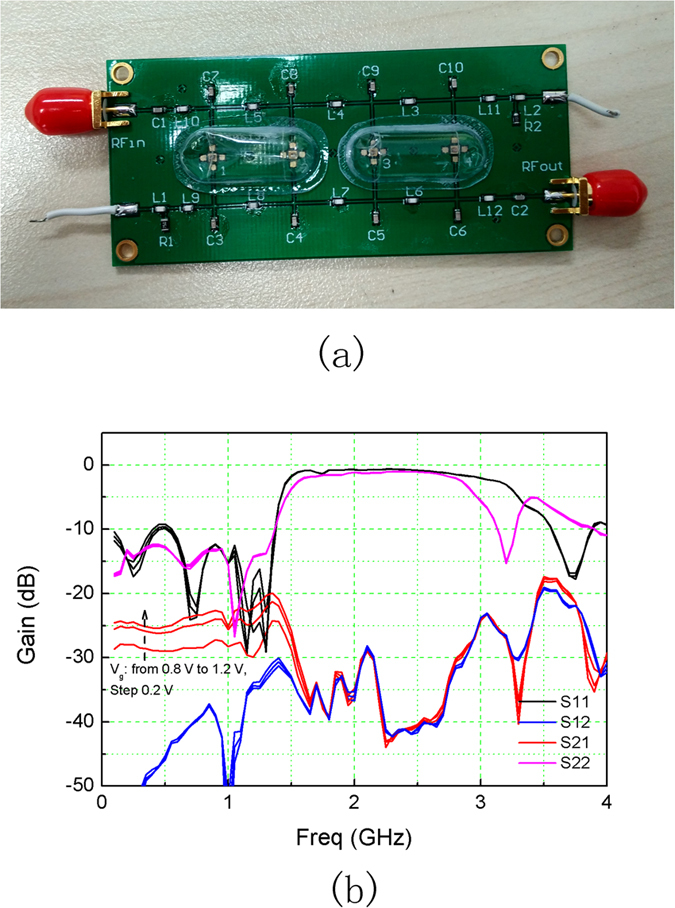
PCB graphene distributed amplifier. (**a**) Photograph of the graphene distributed amplifier. (**b**) Measured S-parameters.

A PCB-level graphene distributed amplifier was fabricated according to Simulation #4, which featured approximately 5 cm × 3 cm dimension, as shown in Fig. 6a. It used FR4 laminate. The capacitors and inductors were in 0805 package. Indidivual GFETs were cut out of the chip and bonded to the PCB board. The bonding wires were limited to about 2 mm, which introduced about 1 nH/mm parasitic effects. After calibration with the short-open-load-through (SOLT) process to eliminate the parasitic effects of the wiring, Agilent N8230C network analyzer were used to characterize the distributed amplifier sample up to 10 GHz. The drain line was biased at 1 V and gate voltage varied from 0.8 V to 1.2 V. The DC biases were induced through AC block inductors.

Test results are shown in Fig. 6b. Maximum gain of −20 dB and bandwidth of 1.5 GHz were obtained. Even though large discrepencies exist, measurement results reassemble the simulation #4. The S21 is dependent on the gate bias V_g_. It increases with each GFET’s transconductance. The charateristic impedences of the artificial transmission lines are approximated as 
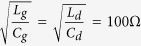
, which are close to 50 Ω and therefore reduce return loss at the source and drain terminals. Several reasons deteriorates the performance as compared to simulation results. Distribution effect took place at GHz frequencies, considering the PCB laminate’s size comparable to the corresponding wavelength. Besides, the bonding wires have not yet been considered in the simulations.

Even though the fabricated work somewhat reassembles a real amplifier, future graphene distributed amplifers should be realized in IC technologies, where precise models of GFETs and passive components should be addressed. Besides, on-chip waveguides could be considered to replace artifical transmission lines. They might further reduces losses.

Distributed amplifiers over the past decades have successfully been applied in compound semiconductor and CMOS technologies^23^,^24^,^25^,^26^. Their application in graphene, a candidate for next-generation semiconductor technology, is especially promising. It conquers graphene’s longstanding problem of being hard to generate gains. In this work, simulations of graphene distributed amplifiers were first performed. A four-stage graphene distributed amplifer based on measured S-parameter of GFETs achieved 4 dB gain and 3.5 GHz bandwidth. We predict that these simulations could be realized by future graphene IC techonologies with precise models. A PCB graphene distributed amplifier was fabricated with large passive components as a proof of concept. Test results of the sample reassembled the corresponding simulation, in which the bandwidth maintained and the gain was sacrificed. The domonstrated works illustrate the principle of graphene distributed amplifiers and proves their feasiblity in future mature IC processes. It is of signicance to the graphene electronics community.

## Methods

### Graphene Synthesis

Graphene in this work was synthesized by CVD method on Pt foils as previously reported. Large scale monolayer graphene films were grown on 180 μm thick Pt foils (99.9 wt % metal basis, 10 mm × 10 mm) by ambient-pressure chemical vapor deposition (APCVD) method. The growth temperature was 1000 °C and CH_4_/H_2_ flow rates were set at 4.5/500 sccm. After growth, Pt foils were quickly pulled out of the high temperature area^27^,^28^. Electrochemical delamination in NaOH solution, the so called “bubbling” method^28^, was used to transfer graphene on a die-by-die basis, limited by the maximum size of the Pt foil.

### GFET Fabrication

The fabrication was based on 200 mm CMOS platform. Conventional BEOL processes fabricated buried gate/source/drain structures made of W. HfO_2_ with equivalent oxide thickness (EOT) of 2 nm was deposited as the gate oxide by atomic layer deposition (ALD) method. After graphene transfer, the channel of the GFETs was defined by contact photolithography and the residual graphene was removed by oxygen plasma etching. The source/drain contact was defined by electron-beam-lithography (EBL). Due to misalignment, certain source-gate and drain-gate overlaps existed in this patch of fabrication. The contacts were formed by lift-off process. The source/drain regions underwent 5 min UVO treatment before the sputtering of 40 nm Pt. The high work function of W induced more doping to graphene and UVO treatment enhanced the metal-graphene’s binding to each other. TML pattern characterization showed the lowest contact resistance of 500 Ωμm.

### Circuit Simulation in Agilent ADS

The Advanced Design System software was version 2012.08. In optimization toolset, the simulated anealing algorithm was used. Three goals were typically used: 1) S21 > 5 dB; 2) S11 < −10 dB; 3) S22 < −10 dB, in the frequency span from 500 MHz to 3 GHz. The weight of the each goals could be independently swiched.

## Additional Information

**How to cite this article**: Lyu, H. *et al.* Graphene Distributed Amplifiers: Generating Desirable Gain for Graphene Field-Effect Transistors. *Sci. Rep.* **5**, 17649; doi: 10.1038/srep17649 (2015).

## Figures and Tables

**Table 1:** Variables in Simulation #1 and #2.

	T1	T2	T3	T4	C_g_	C_d_	L_g_	L_d_
Simulation #1	GFET #4	GFET #4	GFET #4	GFET #4	0.01 pF	0.7 pF	105 nH	7.6 nH
Simulation #2	GFET #1	GFET #2	GFET #3	GFET #4	0.01 pF	1.2 pF	86 nH	7.2 nH
	R_1_	R_2_	Source Impedance	Load Impedance				
Simulation #1	1280 Ω	78.5 Ω	1280 Ω	78.5 Ω				
Simulation #2	1280 Ω	58 Ω	1280 Ω	58 Ω				

**Table 2:** Variables in Simulation #3 and #4.

	T1	T2	T3	T4	C_g_	C_d_	L_g_	L_d_
Simulation #3	GFET #1	GFET #2	GFET #3	GFET #4	1 pF	1 pF	9.8 nH	8.5 nH
Simulation #4	GFET #1	GFET #2	GFET #3	GFET #4	1 pF	1 pF	10 nH	10 nH
	R_1_	R_2_	Source Impedance	Load Impedance				
Simulation #3	73.1 Ω	68.5 Ω	50 Ω	50 Ω				
Simulation #4	100 Ω	75 Ω	50 Ω	50 Ω				

## References

[b1] Wu Y., Farmer D. B., Xia F. & Avouris P. Graphene electronics: Materials, devices, and circuits. Proceedings of the IEEE 101, 1620–1637 (2013).

[b2] Fiori G. *et al.* Electronics based on two-dimensional materials. Nature nanotechnology 9, 768–779 (2014). 10.1038/nnano.2014.207 25286272

[b3] Schwierz F. Graphene transistors. Nature nanotechnology 5, 487–496 (2010). 10.1038/nnano.2010.89 20512128

[b4] Wu Y. *et al.* State-of-the-art graphene high-frequency electronics. Nano Letters 12, 3062–3067 (2012). 22563820 10.1021/nl300904k

[b5] Guo Z. *et al.* Record maximum oscillation frequency in C-face epitaxial graphene transistors. Nano letters 13, 942–947 (2013). 23418924 10.1021/nl303587r

[b6] Wu Y. *et al.* High-frequency scaled graphene transistors on diamond-like carbon. Nature 472, 74–78 (2011). 21475197 10.1038/nature09979

[b7] Liao L. *et al.* High-speed graphene transistors with a self-aligned nanowire gate. Nature 467, 305–308 (2010). 20811365 10.1038/nature09405 PMC2965636

[b8] Lin Y. M. *et al.* 100-GHz transistors from wafer-scale epitaxial graphene. Science 327, 662–662 (2010). 20133565 10.1126/science.1184289

[b9] Cheng R. *et al.* High-frequency self-aligned graphene transistors with transferred gate stacks. Proceedings of the National Academy of Sciences 109, 11588–11592 (2012). 10.1073/pnas.1205696109 PMC3406869 22753503

[b10] Wang H., Nezich D., Kong J. & Palacios T. Graphene frequency multipliers. Electron Device Letters 30, 547–549 (2009).

[b11] Parrish K. N. & Akinwande D. Even-odd symmetry and the conversion efficiency of ideal and practical graphene transistor frequency multipliers. Applied Physics Letters 99, 223512 (2011).

[b12] Lv H. *et al.* Inverted process for graphene integrated circuits fabrication. Nanoscale 6, 5826–5830 (2014). 24745037 10.1039/c3nr06904d

[b13] Wang H., Hsu A., Wu J., Kong J. & Palacios T. Graphene-based ambipolar RF mixers. Electron Device Letters 31, 906–908 (2010).

[b14] Yang X., Liu G., Balandin A. A. & Mohanram K. Triple-mode single-transistor graphene amplifier and its applications. ACS Nano 4, 5532–5538 (2010). 20939515 10.1021/nn1021583

[b15] Lee S., Lee K., Liu C. H., Kulkarni G. S. & Zhong Z. Flexible and transparent all-graphene circuits for quaternary digital modulations. Nature communications 3, 1018 (2012). 10.1038/ncomms2021 22910364

[b16] Habibpour O., Vukusic J. & Stake J. A 30-GHz integrated subharmonic mixer based on a multichannel graphene FET. IEEE Transactions on Microwave Theory and Techniques 61, 841–847 (2013).

[b17] Lin Y. M. *et al.* Wafer-scale graphene integrated circuit. Science 332, 1294–1297 (2011). 21659599 10.1126/science.1204428

[b18] Moon J. S. *et al.* Graphene FETs for zero-bias linear resistive FET mixers. IEEE Electron Device Letters 34, 465–467 (2013).

[b19] Lyu H. *et al.* Double-Balanced Graphene Integrated Mixer with Outstanding Linearity. Nano letters. 10.1021/acs.nanolett.5b02503 (2015). 26378374

[b20] Andersson M. A., Habibpour O., Vukusic J. & Stake J. 10 dB small-signal graphene FET amplifier. Electronics letters 48, 861–863 (2012).

[b21] Guerriero E. *et al.* Graphene audio voltage amplifier. Small 8, 357–361 (2012). 10.1002/smll.201290016 22287083

[b22] Han S. J., Garcia A. V., Oida S., Jenkins K. A. & Haensch W. Graphene radio frequency receiver integrated circuit. Nature communications 5 (2014). 10.1038/ncomms4086 24477203

[b23] Ayasli Y. *et al.* A monolithic GaAs 1-13-GHz traveling-wave amplifier. IEEE Transactions on Microwave Theory and Techniques 30, 976–981 (1982).

[b24] Niclas K. B. *et al.* On theory and performance of solid-state microwave distributed amplifiers. IEEE Transactions on Microwave Theory and Techniques 31, 447–456 (1983).

[b25] Majidi-Ahy R. *et al.* 5–100 GHz InP coplanar waveguide MMIC distributed amplifier. IEEE Transactions on Microwave Theory and Techniques 38, 1986–1993 (1990).

[b26] Ballweber B. M., Ravi G. & David J. A. A fully integrated 0.5–5.5 GHz CMOS distributed amplifier. IEEE J. Solid-St. Circ. 35, 231–239 (2000).

[b27] Xiao K., Wu H., Lv H., Wu X. & Qian H. The study of the effects of cooling conditions on high quality graphene growth by the APCVD method. Nanoscale 5, 5524–5529 (2013). 23674269 10.1039/c3nr00524k

[b28] Gao L. *et al.* Repeated growth and bubbling transfer of graphene with millimetre-size single-crystal grains using platinum. Nature communications 3, 699 (2012). 10.1038/ncomms1702 PMC3293422 22426220

[b29] Lee J. *et al.* Multi-finger flexible graphene field effect transistors with high bendability. Applied Physics Letters 101, 252109 (2012).

[b30] Wang L. *et al.* Negligible environmental sensitivity of graphene in a hexagonal boron nitride/graphene/h-BN sandwich structure. ACS Nano 6, 9314–9319 (2012). 23009029 10.1021/nn304004s

[b31] ZurutuzaáElorza A. Highly air stable passivation of graphene based field effect devices. Nanoscale 7, 3558–3564 (2015). 25631337 10.1039/c4nr07457b

[b32] Dröscher S. *et al.* Quantum capacitance and density of states of graphene. Applied Physics Letters 96, 152104 (2010).

[b33] Xu H. *et al.* Quantum capacitance limited vertical scaling of graphene field-effect transistor. ACS Nano 5, 2340–2347 (2011). 21323320 10.1021/nn200026e

